# Effects of Anticoccidial Vaccination and *Taraxacum officinale* Extract on the Growth Performance, Biochemical Parameters, Immunity, and Intestinal Morphology of *Eimeria*-Challenged Chickens

**DOI:** 10.3390/life13091927

**Published:** 2023-09-17

**Authors:** Anna Arczewska-Włosek, Sylwester Świątkiewicz, Ewa Tomaszewska, Siemowit Muszyński, Piotr Dobrowolski, Damian Józefiak

**Affiliations:** 1 Department of Animal Nutrition and Feed Science, National Research Institute of Animal Production, 32-083 Balice, Poland; sylwester.swiatkiewicz@iz.edu.pl; 2 Department of Animal Physiology, Faculty of Veterinary Medicine, University of Life Sciences in Lublin, 20-950 Lublin, Poland; ewarst@interia.pl; 3 Department of Biophysics, Faculty of Environmental Biology, University of Life Sciences in Lublin, 20-950 Lublin, Poland; siemowit.muszynski@up.lublin.pl; 4 Department of Functional Anatomy and Cytobiology, Faculty of Biology and Biotechnology, Maria Curie-Sklodowska University, 20-033 Lublin, Poland; piotr.dobrowolski@umcs.lublin.pl; 5 Department of Animal Nutrition, Faculty of Veterinary Medicine and Animal Science, Poznań University of Life Sciences, 60-637 Poznań, Poland; damian.jozefiak@up.poznan.pl

**Keywords:** anticoccidial vaccine, dandelion, *Eimeria* challenge, broiler chickens

## Abstract

A total of 160 Ross 308 male chickens were used in a 2 × 2 factorial design to examine the effects of anticoccidial vaccination (ACV; lack or 1× dose recommended by the manufacturer) and dietary supplementation with *Taraxacum officinale* (dandelion) extract (DE; with or without) on growth performance, immunity, biochemical parameters, and intestinal morphology in broiler chickens challenged with *Eimeria* spp. At 20 days of age, all birds were challenged with a 25× dose of ACV, including *Eimeria acervulina*, *E. maxima*, *E. mitis*, and *E. tenella*. No interaction between ACV and DE was observed in terms of growth performance. Vaccinated birds showed increased feed intake (FI) and feed conversion ratio (FCR) during the 11–20 day period. Meanwhile, DE supplementation led to decreased FI and body weight gain (BWG) during the 1–10 day period. ACV effectively induced immunity against *Eimeria*, as evidenced by reduced oocyst shedding and less intestinal lesions, decreased levels of pro-inflammatory interleukin-6, and improved BWG during both the post infection (PI) period (21–35 days) and the entire growth period. DE supplementation lowered FCR and increased BWG during the 35–42 day period, increased the concentration of butyric acid in the cecal digesta, and lowered oocyst shedding PI. In vaccinated birds, DE elevated levels of plasma total protein and immunoglobulin M, and influenced tight junction proteins zonula occludens-1 and claudin-3, indicating a more robust epithelial barrier. DE also lowered alanine aminotransferase activity in unvaccinated birds. Both ACV and DE independently improved intestinal morphology in the jejunum, decreasing crypt depth and increasing the villus height-to-crypt ratio. These findings suggest that both ACV and DE could be effective strategies for managing coccidiosis in broiler chickens.

## 1. Introduction

Coccidiosis, caused by *Eimeria* protozoan parasites, is a prevalent disease with global economic implications for broiler chickens [1,2]. The global poultry sector faced significant financial burdens due to this disease, with losses estimated at GBP 10.4 billion in 2016 [3]. These losses arise from factors such as reduced weight gain, decreased feed conversion efficiency, increased medication costs, and increased mortality [3,4,5]. In cases of coccidiosis, damage to the intestinal mucosa—accompanied by oocyst proliferation—results in symptoms like diarrhea, malabsorption, and inflammation. It may also predispose chickens to dysbacteriosis, including the development of *Clostridium perfringens*-mediated necrotic enteritis [5,6,7]. To mitigate the impact of coccidiosis, anticoccidial drugs and anticoccidial live vaccines (ACVs) have been employed as the primary control strategies [4,8,9].

Recently, there has been growing interest in the use of ACVs for broilers, driven by the emergence of *Eimeria* strains resistant to anticoccidials [10]. The ACVs contain either attenuated or live wild-type strains of *Eimeria* and induce a protective immune response in vaccinated birds [9]. These vaccines are typically administered orally—through spraying, gel, or drinking water—making it feasible for mass administration to large flocks of broilers [6]. They stimulate both cellular and humoral immune responses, effectively mimicking the infection process without causing severe disease. While considered an effective approach for controlling coccidiosis in layers and breeding flocks, ACVs present some concerns when used in broilers. The primary issue is the potential for vaccine-induced pathogenicity, as ACVs may occasionally trigger mild to moderate symptoms of coccidiosis or serve as a predisposing factor for bacterial enteritis in vaccinated birds. Such outcomes can affect bird performance, which may not be fully compensated for given the relatively short lifespan of birds. Additionally, the immunity developed is species-specific; hence, birds may remain susceptible to other *Eimeria* species not included in the vaccine [4,9,11,12,13,14,15].

In light of these challenges, there is a growing interest in exploring natural methods that can support coccidiosis immunoprophylaxis and improve the overall health and productivity of vaccinated broiler chickens. One promising strategy is the use of plant extracts in poultry nutrition as they serve as a rich source of diverse bioactive substances capable of exerting their beneficial effects through various mechanisms of action [16,17,18,19,20].

Dandelion (*Taraxacum officinale*), a herbaceous plant in the Asteraceae family, is recognized for its various medicinal properties, including anti-inflammatory, antibacterial, antioxidant, hepatoprotective, anticancer, and immunomodulatory effects [21]. The primary bioactive ingredients of dandelion are phenolic compounds, terpenes, carbohydrates, proteins, fatty acids, vitamins, and minerals. Among its antioxidant and anti-inflammatory phenolic compounds, derivatives of hydroxycinnamic acid—specifically chicoric acid, chlorogenic acid, and caffeic acid—have been found in the highest concentrations [22,23]. Various studies have reported dandelions’ ability to improve growth performance in poultry [21,24,25,26]. A recent study demonstrated that dandelion enhances feed efficiency in broilers by enhancing intestinal barrier function, decreasing proinflammatory cytokines, and optimizing the composition of the intestinal microbiota [25].

Thus, it was hypothesized that dandelion extract (DE) could effectively mitigate the potential negative effects of ACV during the development of ACV-induced immunity to coccidiosis, especially concerning the deterioration of growth parameters. Furthermore, when birds are exposed to *Eimeria* infections, DE has the potential to either enhance the vaccine’s efficacy or alleviate the adverse effects of the infection in unvaccinated birds.

## 2. Materials and Methods

### 2.1. Experimental Design, Birds, and Diets

Animal testing procedures received approval from the second Local Ethics Committee for Animal Testing in Krakow, Poland. The experiment followed a 2 × 2 factorial arrangement resulting in four treatment groups. Each treatment group had five replicates (pens), with each pen housing eight male Ross 308 chicks. Experimental factors included either the absence or presence of a single dose of ACV Paracox^®^-5 (MSD Animal Health, Milton Keynes, UK) administered at 1 d of age. This was combined with either the presence or absence of dietary supplementation with *T. officinale* extract, also known as dandelion (Herberry Ltd., Stawiguda, Poland).

In total, 160 1-day-old male Ross 308 broiler chickens were purchased from a commercial hatchery (H and P Hatching and Poultry Breeding, Orzesze-Gardawice, Poland) and randomly allotted to different treatment groups at 1 d of age. The experiment lasted for 42 days. Chickens were maintained under conventional environmental conditions, with initial temperatures set at 32 °C on day 1, gradually decreasing to 21 °C by day 21. They were housed in floor pens, each offering 0.76 m^2^ of space and equipped with two nipple–cup drinking systems and one trough feeder. Wood shavings were employed as bedding to facilitate the recirculation of vaccine-originated oocysts. To minimize the transfer of oocysts and reduce the risk of contamination among unvaccinated groups, PVC sheet barriers were installed between the pens.

The birds had ad libitum access to water and feed. The basal diets were formulated to meet or exceed the nutritional standards for broilers during various feeding phases: starter (1–10 days), grower 1 (11–19 days), grower 2 (20–35 days), and finisher (36–42 days) (Table 1) [27]. The experiment was conducted at the broiler facility within the Experimental Station in Aleksandrowice, operated by the National Research Institute of Animal Production in Poland. This facility maintains a stringent sanitation regimen to mitigate challenges that broilers might encounter in large-scale intensive production systems. To make the diets slightly challenging, wheat and rye (as sources of nonstarch polysaccharides) were incorporated, along with fishmeal. Diets for vaccinated birds were free of in-feed coccidiostats, which is crucial for allowing the development of acquired immunity and the recirculation of vaccine strains. In contrast, feed mixtures for unvaccinated birds provided up to the 20th day of age were supplemented with salinomycin (70 ppm; Sacox 120; Huvepharma, Antwerp, Belgium). The administration of coccidiostats in the diet of unvaccinated groups, along with the high level of sanitation, aimed to prevent the potential transmission of vaccine oocysts and their subsequent proliferation in the avian gastrointestinal tract. This setup allowed the evaluation of the effects of dandelion extract under conditions of either first-time or repeated exposure of the chickens to *Eimeria* spp.

At 20 d of age, all birds were challenged with *Eimeria* spp. through individual oral administration of a 25-fold dose of ACV Paracox^®^-5. The vaccine’s 25-fold dose (0.1 mL) was suspended in 0.24 mL of distilled water and given orally. The composition of this vaccine is detailed in Section 2.2. The attenuated vaccine dose used for the infection was not expected to induce clinical coccidiosis.

### 2.2. Experimental Factors

At 1 day of age, half of the chickens received an oral administration of a single recommended dose of the live attenuated ACV, Paracox^®^-5 (MSD Animal Health, Milton Keynes, UK) before being placed in their designated pens. Each vaccine dose contained sporulated oocysts of various *Eimeria* species in the following amounts: *E. acervulina* 500–600, *E. maxima* CP 200–230, *E. maxima* MFP 100–130, *E. mitis* HP 1000–1300, and *E. tenella* HP 500–650. The vaccine dose (0.004 mL) was suspended in 0.24 mL of distilled water and given orally. An equivalent volume of distilled water was administered to the unvaccinated groups of birds.

In the treatment groups intended for obtaining *T. officinale* extract (Herberry Ltd., Stawiguda, Poland), a feed additive was incorporated into the basic feed mixtures at a dose of 2 g/kg of feed. The DE was prepared from the radix and herba of *T. officinale* using the following method: an aqueous extract was obtained at a plant-to-water ratio of 1:10 (DER 1:10). This extract was then concentrated 10-fold using a rotary evaporator under reduced pressure. The concentrated extract was subsequently blended with maltodextrin in a ratio of 2726 g of extract to 700 g of maltodextrin. This mixture was then spray-dried, yielding 1002 g of powder. The concentration of chicoric acid, the major phenolic compound in *T. officinale*, was determined in the final product. Quantitative analysis of chicoric acid was performed using a PerkinElmer Altus™ HPLC system equipped with a Diode Array Detector (DAD) (PerkinElmer, Shelton, CT, USA) utilizing detection wavelength of 330 nm. Chromatographic separation was achieved on a LiChrospher 100, C18, 5 µm column (125 × 4 mm; Merck KGaA, Darmstadt, Germany) maintained at 35 °C. The mobile phase consisted of 0.1% formic acid in ultrapure water and 0.1% formic acid in acetonitrile, with a flow rate of 0.75 mL/min. The injection volume was set at 10 μL and the detection wavelength was 330 nm. The chicoric acid content in the final preparation was determined to be 0.65 g/kg.

### 2.3. Sample Collection and Analytical Procedure

Weekly feed intake (FI) measurements were taken and the broiler weight was recorded on days 1, 10, 20, 35, and 42. Growth performance parameters, including body weight gain (BWG), FI, and the feed conversion ratio corrected for mortality (FCR) were calculated for each feeding phase. These parameters were analyzed on a pen basis (*n* = 5).

To determine oocyst excretion, the concentration of oocysts per gram of feces (OPG) was measured using the McMaster concentration method in a McMaster counting chamber [28]. OPG assessments were carried out on pooled fecal samples collected from each experimental pen (*n* = 5). In the vaccinated groups, these assessments took place on days 7, 14, and 20 postvaccination (PV). In all experimental groups, they were conducted on days 5, 6, 7, and 15 post infection (PI). To achieve a normal distribution, OPG values underwent a logarithmic transformation [log10 (OPG + 1)].

At 25 days of age (5 d PI), five birds per treatment (one bird per replicate pen; *n* = 5) were slaughtered by decapitation after 8 h of fasting and electrical stunning using STZ6 apparatus (Koma Ltd., Wilkanowo, Poland). Blood samples were collected in heparin tubes and the intestines were immediately excised for lesion scoring (LS) due to the *Eimeria* challenge and for histological analysis. Lesions in the duodenum, jejunum/ileum, and ceca were evaluated using the Johnson and Reid method, with a scale ranging from 0 (no visible lesion) to 4 (most severe lesion) [29]. For histological analysis, two 20-mm sections were collected from the duodenum (2 cm behind the gizzard), jejunum (from the midpoint of this intestinal segment), and ileum (5 cm before the ceca). These were preserved in 10% formalin, following standard histological procedures as previously described [30]. After a 24-h fixation period, tissue samples were washed with tap water, dehydrated, embedded in paraffin, and cut into 4 μm sections (Microm HM 360, Microm, Walldorf, Germany). They were then stained with Goldner’s trichrome. Microscopic observations were conducted using optical microscopes (BX63 and CX43, Olympus, Tokyo, Japan). Various histomorphometric parameters, such as villus height (VH) and thickness, crypt depth (CD) and width, as well as the thickness of the mucosa, submucosa, and lamina muscularis (both longitudinal and circular), were measured [31]. The ratio of villus height to crypt depth (VH/CD) for individual pairs of villus and crypt was determined and the mucosal surface absorptive area was calculated as detailed in the previous literature [32]. For each parameter, eight histomorphometry measurements per bird were taken using graphic analysis software (ImageJ 1.51; National Institutes of Health, Bethesda, MD, USA).

The jejunum tissue slices were utilized for immunohistochemical (IHC) staining, following a protocol previously described [30]. To label tight junctions (TJs), rabbit polyclonal antiZO-1 (zonula occludens-1) antibody (orb11587, Biorbyt, St. Louis, MO, USA) and rabbit polyclonal anticlaudin 3 antibody (AB15102; Abcam, Cambridge, UK) were used as the primary antibodies. These sections were subsequently treated with the secondary antibody: peroxidase-conjugated goat antirabbit (#611-1302, Rockland Immunochemicals, Inc., Limerick, PA, USA). The sections were then exposed to 3,30-diaminobenzidine tetrahydrochloride (DAB D5905; Sigma-Aldrich, St. Louis, MO, USA) for chromogenic development and counterstained with Mayer’s hematoxylin (MHS32-1L; Sigma-Aldrich, St. Louis, MO, USA). IHC images were captured using CX43 and BX63 light microscopes (Olympus, Tokyo, Japan) and converted into 8-bit grayscale format. This allowed for the quantitative measurement of the intensity of the immunoreaction (IR) for ZO-1 in villi and claudin-3 in crypts by comparing the optical density (OD) values of the images. OD calculations followed the ImageJ protocol and used a Kodak 3-step calibration tablet in conjunction with ImageJ’s integrated Rodbard function. This translated the 8-bit pixel values into calibrated OD readings [33]. Representative images of the IHC reactions for zonula occludens-1 and claudin-3 on formaldehyde-fixed sections from the jejunum are presented as supplementary material (Figure S1).

The blood samples were centrifuged at 3000× *g* for 10 min and the resulting plasma was analyzed for concentrations of total immunoglobulin Y, M, and A (IgY, IgM, and IgA), tumor necrosis factor α (TNF-α), interleukin-1β (IL-1β), interleukin-6 (IL-6), ceruloplasmin (Cp), and fibrinogen (Fb). Commercial quantitative ELISA kits (FineTest, Wuhan Fine Biotech Co., Ltd., Wuhan, China) were used for these analyses.

Additionally, blood plasma samples were further evaluated for specific biochemical parameters using commercial kits from Cormay Co. (PZ Cormay Inc., Lomianki, Poland). These parameters included the total protein (TP), triacylglycerols (TG), total cholesterol (TC), and glucose (GLU) levels as well as enzyme activities for aspartate aminotransferase (AST), alanine aminotransferase (ALT), alkaline phosphatase (ALP), and lactate dehydrogenase (LDH).

At 42 d of age, two chickens per pen (*n* = 10) were slaughtered following electrical stunning and their cecal digesta were collected and immediately frozen for the determination of volatile fatty acids (VFAs). Upon thawing, a 5 g aliquot of the digesta sample was diluted in 50 mL of water. Subsequently, 5 mL of the filtrate was mixed with 1 mL of 24% sulfuric acid and centrifuged. The supernatant was then used for chromatographic analysis to determine the concentration of cecal VFAs. The analysis was performed using a GC-2010 Plus gas chromatograph (Shimadzu Corp., Kyoto, Japan) equipped with a capillary column (CP-WAX 58 FFAP 25 m × 0.53 mm; Agilent Technologies, Palo Alto, CA, USA).

The basic compound feeds formulated for each feeding phase were analyzed using AOAC standard methods [34]. The analyses included measurements for moisture, crude fat, crude protein, ash, amino acids, calcium, and total phosphorus content, following method 930.15, method 920.39, method 984.13, method 942.05, method 982.30, method 968.08, and method 965.17, respectively.

### 2.4. Statistical Analysis

Data were analyzed using a two-way analysis of variance (ANOVA) through STATISTICA software (version 13.3; StatSoft Inc., Tulsa, OK, USA) to determine the main effects of the treatments. In this analysis, ACV and DE dietary supplementation were considered as the primary influencing factors and potential interactions between them were also evaluated. The effect of DE supplementation on OPG results in vaccinated chickens was assessed using a one-way ANOVA. To identify differences between the treatments, Duncan’s multiple range post hoc test was used. Statistical significance was set at *p* < 0.05.

## 3. Results

### 3.1. Growth Performance

The effects of ACV and DE on basic growth performance results across various feeding phases (1–10 days for the starter phase, 11–20 days for grower 1, 21–35 days for grower 2, 36–42 days for the finisher phase, and 1–42 days overall) are presented in Table 2. No significant interactions between the experimental factors were observed during any of the analyzed periods.

Regarding independent effects, during the starter feeding phase, DE significantly reduced FI, which consequently led to a decrease in BWG. The impact of ACV became noticeable starting from the grower 1 feeding phase. In this phase, ACV significantly increased FI, resulting in increased FCR, although it did not affect BWG. During the same period, DE showed a tendency to increase BWG by 2.2% (*p* = 0.051).

After the experimental challenge with *Eimeria* at 20 days of age, vaccination with ACV led to a significant improvement in BWG during the grower 2 phase and increased FI during the finisher phase. No impact from DE was observed in the grower 2 phase. However, in the finisher phase, supplementation with the extract significantly increased BWG and showed a tendency to lower the FCR by 6.3% compared to the unsupplemented groups. For the entire experimental period, neither treatment showed an effect on FCR. Nevertheless, a significant impact of ACV was noted, resulting in 3.1% and 4,6% increases in BWG and FI, respectively, compared to unvaccinated groups. Additionally, a tendency towards increased BWG was observed in birds supplemented with DE, showing a 2.41% increase compared to unsupplemented groups.

### 3.2. Oocyst Shedding and Lesion Score

**Figure 1:**
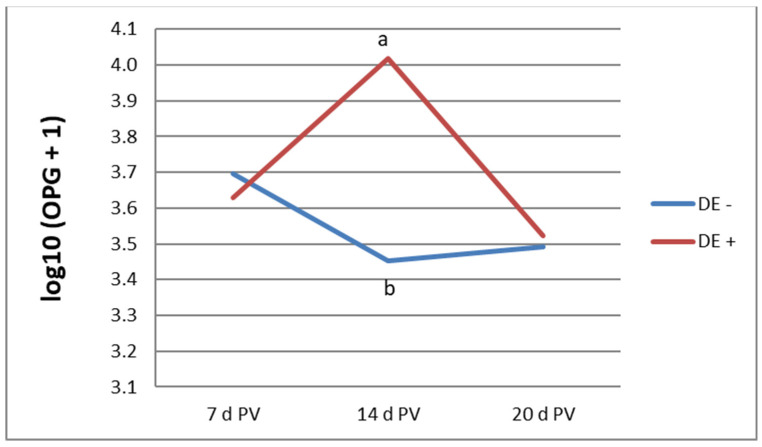
Oocyst output in the vaccinated groups [log10 (OPG + 1)]; a, b—mean values not sharing a common letter are significantly different (*p* ≤ 0.05); OPG—oocysts per gram, pv—postvaccination, and DE—dandelion extract.

Figure 1 illustrates the oocyst shedding profile in vaccinated bird groups at 7, 14, and 20 days PV. Statistically significant differences were observed at 14 days PV, during which birds receiving the diet supplemented with DE shed a higher number of oocysts. By 20 days PV, the OPG values in all vaccinated birds were comparable. No oocysts were detected in unvaccinated groups up to 20 d of age.

The profile of PI oocyst shedding and the results of LS conducted 5 days after *Eimeria* spp. infection are presented in Table 3. A significant interaction between ACV and DE was only recorded in OPG at 5 days PI. During this period, supplementation with DE resulted in a decrease in OPG, with no effect observed in unvaccinated groups. In terms of independent effects, vaccinated birds shed significantly fewer oocysts at 5, 6, and 7 d PI. Supplementation with DE lowered OPG at 7 and 15 d PI, regardless of vaccination status. During LS, no cecal lesions were detected in any of the groups. A significant effect of lowered LS in the duodenum and a tendency (*p* = 0.059) toward lowered LS in the jejunum and ileum were observed. DE supplementation did not affect LS at 5 d PI.

### 3.3. Intestinal Morphology and Epithelial Barrier Characteristics

The results of histomorphometry measurements for the duodenum, jejunum, and ileum are detailed in Table 4, Table 5 and Table 6, respectively. No significant interactions between ACV and DE were observed in any section of the intestine.

Concerning independent effects, neither ACV nor DE had a significant impact on the morphology of the duodenum.

In the jejunum, ACV significantly increased villus thickness and the VH/CD ratio and reduced CD by 17.2%, 17.6%, and 15.6%, respectively. DE supplementation also led to a 19.2% increase in villus thickness.

In the ileum, the effect of ACV diverged; vaccinated birds displayed deeper crypts and a reduced VH/CD ratio by 25.0% and 20.6%, respectively, compared to unvaccinated birds. Additionally, the ileum’s longitudinal muscularis lamina in birds supplemented with DE was 17.8% thinner than that in unsupplemented chickens.

The results of the quantitative analysis examining the intensity of zonula occludens-1 (ZO-1) and claudin-3 IR in the jejunum of broiler chickens at 5 days post *Eimeria* infection are presented in Table 7. This table presents the OD values for ZO-1 in the villi and claudin-3 in the crypts.

A significant interaction between ACV and DE was observed for both ZO-1 and claudin-3 (*p* ≤ 0.001 for both) in addition to a significant independent effect of ACV.

In vaccinated birds, DE supplementation led to higher OD values, signifying stronger ZO-1 IR in the villi. Conversely, in unvaccinated birds, DE supplementation was associated with lower OD values for ZO-1 IR.

Regarding claudin-3, dietary DE supplementation was associated with lower OD values, indicating weaker IR in vaccinated birds. However, it did not affect this parameter in unvaccinated birds.

### 3.4. Immunoglobulin, Cytokines, and Acute Phase Protein Levels

The effects of the experimental factors on plasma immunoglobulin, cytokines, and acute phase protein levels in broiler chickens at 5 days post *Eimeria* spp. infection are presented in Table 8.

A significant interaction between ACV and DE (ACV × DE) was observed for IgY. However, post hoc analysis showed that the only significant difference occurred between the group of unvaccinated birds supplemented with DE and the group of vaccinated birds also receiving DE in their diet.

Similarly, a significant interaction between ACV and DE was noted for IgM. The highest IgM levels were observed in vaccinated chickens that were also supplemented with DE whereas DE had no effect on IgM levels in unvaccinated birds.

For IL-6, a significant effect of ACV was evident as vaccinated chickens displayed IL-6 levels that were 19.4% lower than those found in unvaccinated birds.

For IL-1β, a trend toward lower levels was observed in broilers supplemented with DE, irrespective of their vaccination status (*p* = 0.076).

Regarding IgA, TNF-α, Cp, and Fb, neither ACV nor DE demonstrated significant effects, nor were any significant interactions between these factors detected.

### 3.5. Biochemical Parameters

The effects of the experimental factors on the biochemical indices of chicken blood collected at 5 days PI are summarized in Table 9. While all parameters were within the normal range for poultry, some were significantly affected by the experimental treatments. A significant interaction between ACV and DE was noted for ALT, TP, and TC levels.

In the case of ALT, vaccinated birds exhibited relatively low activity levels that were not influenced by DE supplementation. However, for unvaccinated birds showing the highest ALT activity, DE supplementation led to a reduction in ALT activity, bringing it to a level similar to that observed in vaccinated groups.

For TP, an increased level was noted in birds that were both vaccinated and supplemented with DE. In contrast, DE did not affect this parameter in unvaccinated birds.

For TC, a significant ACV × DE interaction was also observed. The highest TC concentration was found in groups that were both vaccinated and DE-supplemented, compared to either of the unvaccinated groups.

No significant independent effects of ACV or DE, nor any significant interactions between these factors, were detected for the activities of AST, LDH, ALP, or TG and GLU concentrations.

### 3.6. Volatile Fatty Acids in Cecal Digesta

The effects of experimental factors on the concentrations of VFAs in the cecal digesta of broiler chickens at 42 days of age are presented in Table 10. The dominant VFA was acetic acid, followed by butyric and propionic acids.

No significant interactions between ACV and DE were noted for any of the VFAs. Similarly, neither ACV nor DE showed significant effects on the concentrations of acetic, propionic, isobutyric, isovaleric, valeric, branched-chain fatty acids (BCFAs), or total VFAs. However, a significant effect of DE was observed for butyric acid. Chickens supplemented with DE exhibited a significantly higher concentration of butyric acid (by 27%) in the cecal digesta compared to unsupplemented chickens.

## 4. Discussion

Immunoprophylaxis with anticoccidial vaccines offers several advantages over conventional chemoprophylaxis. These include reduced reliance on anticoccidial drugs, which help to mitigate concerns about drug residues in poultry products and the potential development of drug-resistant parasites [9].

The deterioration of growth performance is a major concern for poultry producers contemplating the use of immunoprophylaxis with live anticoccidial vaccines. According to vaccine manufacturers, immunity should be established by 14 days PV through exposure to vaccine-derived oocysts. These oocysts initially administered through a vaccine dose undergo a developmental cycle in the intestinal epithelial cells. Subsequently, the resulting progeny oocysts are excreted into the environment where they sporulate and are later ingested by birds. For effective immunity development, 2–3 such cycles within the intestinal epithelium are necessary [35]. It is crucial to avoid the use of anticoccidials or other feed additives with coccidiostatic properties during this period. Such additives could interfere with the developmental cycle of vaccine strains, thereby impacting immunity development and potentially diminishing the efficacy of vaccination in the event of a coccidiosis outbreak.

Our analysis of oocyst shedding up to 20 days PV revealed that dietary supplementation with DE did not negatively impact the proliferation of vaccine-derived oocysts. In fact, at 14 days PV, we noted even higher oocyst counts in the group of chickens receiving DE compared to those that were unsupplemented. Oocyst proliferation in the intestinal epithelium often disrupts intestinal integrity, impairs nutrient absorption, and induces inflammation. These changes can negatively impact production parameters during this period [5,6,7]. In the current study, in vaccinated birds we observed increases in FI and FCR without affecting BWG at 20 days of age. It is presumed that, after achieving immunity to coccidiosis, the birds initiated a recovery phase and activated compensatory mechanisms. Previous studies also noted a decline in performance during the first 2–3 weeks after ACV [36,37,38]. Similar to our findings, Das et al. [39] reported that bird performance was particularly affected during the growing phase from 10 to 20 days of age.

In subsequent growing periods after the infection, no deterioration in growth performance was observed among the vaccinated birds. Moreover, the current study affirmed the efficacy of the applied anticoccidial vaccine, Paracox^®^-5. Vaccinated birds exhibited higher BWG PI, which can be linked to significantly lower oocyst counts in the feces and reduced LS in the small intestine. The significant impact of ACV was also evident in increased BWG for the entire experimental period. The effectiveness of ACVs in inducing immunity and protecting against subsequent *Eimeria* infections, as well as mitigating their adverse effects on performance, is well-documented. This is evidenced by increased BWG, reduced FCR, and fewer intestinal lesions in vaccinated chickens compared to unvaccinated ones [40,41]. Even if temporary performance setbacks were observed in the first two weeks of PV [42], the overall efficacy of ACVs was confirmed. However, reports suggest that the negative impact of ACV on growth performance in infected chickens can persist until the end of the rearing period. This discrepancy may arise from differences in the species composition of the vaccine and the inoculum used during infection [36]. In such cases, ACV fails to offer complete protection against the challenge, as resistance to *Eimeria* is species-specific.

No interaction between ACV and DE was observed in any of the experimental periods with respect to growth parameters. During the starter feeding phase, DE significantly reduced FI, which correspondingly led to a decrease in BWG in both vaccinated and unvaccinated groups. In the study by Mao et al. [25], administering 500 mg of dried dandelion per kg of feed led to reduced FI but resulted in a lowered FCR of up to 21 d of age. However, when the dandelion dose was increased to 1000 mg/kg, this effect was not observed. Qureshi et al. [26] reported that dietary supplementation with 0.5% dried dandelion leaves did not impact FI but did reduce FCR and improve BWG for birds aged 1–3 weeks. In the current study, the negative effects of DE were not observed from day 11–20 or thereafter, which could suggest that the applied dose was too high for chicks during the early rearing period. It is worth noting that dandelion is considered a plant with very low toxicity [43]. From another perspective, Yang et al. [38] suggested that the administration of essential oils (EOs) may be more beneficial starting from the grower phase rather than the starter phase given that the gut microbiota is not yet fully established and stable at that early stage.

In the finisher phase, DE supplementation significantly increased BWG and showed a tendency to reduce FCR in our study. This suggests that DE supplementation may have a more pronounced effect on growth performance during later stages and supports the idea of a recovery phase after the onset of infection.

A significant positive effect of DE on decreased oocyst shedding was also found at 7 and 15 days PI. Oocyst output, along with growth performance and LS, is considered one of the crucial criteria for assessing vaccine efficacy [6]. In vaccinated birds, DE supplementation resulted in fewer oocysts being shed at 5 days PI. This outcome may be attributable to the immunomodulatory effects of DE, which enhanced the immune response of the vaccinated birds, thereby increasing their resistance to *Eimeria* spp. infection. This was reflected in elevated levels of IgY and IgM in vaccinated birds receiving DE in their diet. However, it is important to note that the immunoglobulins measured were not specific to *Eimeria* but rather represented total antibody levels for the particular class. The cellular response is generally considered to be of greater importance in natural immunity against *Eimeria* infection and serum antibody levels are thought to be of lesser relevance for immune protection following such an infection [6].

During coccidiosis, the intestinal epithelial tissue suffers damage as oocysts invade and proliferate within intestinal cells, destroying them in the process. Common adverse effects of *Eimeria* infection on intestinal morphology include a decrease in VH, deeper crypts, and a lower VH/CD ratio. Shorter villi, which are associated with a smaller absorptive area, can lead to malabsorption. Meanwhile, deeper crypts indicate rapid tissue turnover and an increased need for new tissue [44]. In the present study, both vaccination and DE significantly improved morphometric parameters in the jejunum. ACV and DE independently increased villus thickness while vaccinated birds also exhibited shallower crypts and a lower VH/CD ratio. However, in the ileum, ACV led to deeper crypts and a reduced VH/CD ratio without affecting VH. This effect may represent an adaptive mechanism for intestinal epithelial recovery, enhancing cell proliferation and boosting enterocyte turnover following damage by *Eimeria* development. The use of DE as a dietary supplement was also found to significantly thin the muscularis longitudinal layer, potentially due to a reduction in the accumulation of inflammatory cells. Healthy broilers fed a diet containing 0.5% dandelion showed less cellular infiltration in the mucosa and submucosa compared to the control group, with no effect on villi and crypt architecture [45].

The decrease in the concentration of cecal VFA has been previously reported as a negative effect of *Eimeria* infection, which could be linked with a shift in microbiota [7,46]. In the current study, neither the concentrations of individual nor total VFAs in cecal digesta were affected by ACV, DE, or their interaction, except for an increase in butyric acid in both groups of birds receiving DE in the diet. However, it is important to note that the cecal digesta were collected after the growth test at 42 d of age. Any potential differences between treatments that might have occurred directly after the infection at 20 d of age may no longer be visible.

The increase in butyrate levels in DE-supplemented groups points to the positive impact of this phytogenic additive on shifting the microbiota towards butyrate-producing species. An elevated level of butyric acid in ileal digesta, along with an affected microbiota profile, has also been previously observed in broilers given dandelion in their diet [25]. This metabolite of microbial fermentation is not only an energy source for enterocytes but also contributes to various other functions. These include gut tissue development, gene expression regulation, and cell differentiation, as well as immune and microbial modulation. Furthermore, it plays a role in reducing oxidative stress and controlling diarrhea [47].

Under healthy conditions, intestinal epithelial TJs effectively prevent the paracellular entry of harmful substances and antigens from the gastrointestinal tract, such as bacteria, toxins, and degraded food components [48,49]. However, in conditions like *Eimeria* infection, the integrity of the intestinal barrier is compromised, leading to increased permeability to luminal antigens. This “leakiness” allows foreign antigens to penetrate the intestinal tissue [50]. Antigen-presenting cells and T-lymphocytes process these antigens, triggering an inflammatory response. In coccidiosis, there is increased synthesis of proinflammatory cytokines such as IL-1β, IL-6, and TNF-α [51], which further disrupt the TJ barrier and amplify the inflammatory process by allowing more luminal antigens to permeate [48]. There are conflicting reports regarding the response of TJs to *Eimeria* infection. In a study with graded *E. maxima* infection in broiler chickens, gene expressions of occludin, ZO-1, claudin-2, and intestinal mucin 2 linearly and quadratically decreased, while the gene expression of claudin-1 was significantly upregulated at 5 days PI. Concurrently, growth performance worsened and gut permeability increased linearly in response to higher levels of *E. maxima* challenges [52]. Castro et al. [53] observed increased gut permeability at 5 days PI in broilers challenged with *Eimeria*. There was also an upregulation of serum claudin-1 gene expression by day 6 PI, while the expressions of ZO-1, ZO-2, claudin-2, and occludin remained unaffected compared to unchallenged birds. On the other hand, Alagbe et al. [54] observed upregulated levels of TNF-α, IL-1β, IL-6, and IL-10 with no affected expression of claudin-1 and occludin at 6 d PI. Similarly, Mohsin et al. [55] did not record any difference in the expression of ZO-1 and claudin-1 between the group challenged with *E. tenella* and the unchallenged group. In our study, at 5 days PI, a significant independent effect of ACV leading to reduced IL-6 levels was observed, along with a trend towards decreased IL-1β in groups supplemented with DE. At the same time, in vaccinated birds, DE supplementation increased the IR intensity for villi ZO-1 but decreased it for claudin-3 IR in the jejunum crypts. In unvaccinated birds, DE reduced ZO-1 IR intensity. ZO-1 is located on the intracellular side of the cell membrane, close to the tight junction strands, and is thought to play a crucial role in tight junction functionality. On the other hand, claudins, a primary component of tight junctions, establish a barrier that regulates paracellular transport within the intestinal epithelium [49]. To our knowledge, as of now, no study on the effect of dandelion on the intestinal barrier in chickens infected with *Eimeria* has been published. However, in healthy broilers, Mao et al. [25] discovered that a 500 mg/kg dandelion supplementation reduced TNF-α levels and enhanced ileal gene expression for TJ proteins, including claudin, occludin-1, and mucin 1. This was associated with improved feed utilization, without affecting ZO-1 expression. Therefore, the variations in the TJ protein profile observed in the current study in challenged birds might be an adaptive response to infection. Furthermore, the modification in TJ protein composition, combined with the proinflammatory cytokine results from our study in vaccinated birds fed DE-supplemented diets, suggests that these birds may have experienced milder effects from the challenge and maintained a better-protected epithelial barrier.

Increased liver enzymatic activity is often observed during coccidiosis [55]. In the current study, DE significantly lowered ALT activity in unvaccinated birds. This aligns with previous information about the hepatoprotective effect of dandelion, which has been shown in healthy birds to positively influence both enzymatic activity modulation [26] and histological liver examinations by reducing Kupffer cell hyperplasia and perivascular mononuclear cell infiltration [45]. Additionally, the absence of a negative impact of the feed additive on liver enzymatic activity suggests that it does not have a toxic interaction at the dose used in the experiment. An increase in plasma total protein levels was also noted in the group of vaccinated birds in the current study. Previous research has documented a decrease in serum TP levels on days 5–7 following infection with *E. acervulina*, *E. tenella*, and *E. maxima* [56]. Thus, higher TP levels may indicate less damage to the intestinal mucosa from infection, leading to better nutrient absorption or reduced leakage into the intestinal lumen. Previous studies on chickens have demonstrated that dandelion reduces the concentrations of TG, TC, and GLU in the blood of uninfected chickens [24,26]. However, such hypoglycemic and hypocholesterolemic effects at 5 d PI were not observed in the current study.

To sum up the current situation, the practical use of live ACVs in broilers is likely to increase given the current trends to minimize the use of chemotherapeutics due to growing parasite resistance and consumer expectations. As third-generation vaccines (subunit vaccines) are still under development and may take a considerable time to enter the market, poultry producers will continue to rely on live vaccines [9]. Although ACVs are generally effective in protecting against severe effects of coccidiosis, there can be temporary deterioration in growth performance PV, which may not be fully compensated for by the end of the rearing period. Moreover, field infections might arise from species not included in the ACV, in which case the vaccine may not offer full protection as immunity to *Eimeria* is species-specific. Therefore, nutritional approaches that support immune system development and mitigate the negative effects on growth performance, while also combating infection, are promising. Phytogenic feed additives are, next to probiotics, the most frequently tested alternatives to standard programs against coccidiosis [16,17,18,19,20,57]. *T. officinale* could be considered a medicinal plant due to its rich content of bioactive substances, which have demonstrated broad pharmacological properties in both in vitro and in vivo studies [21,22,23]. Although this plant is widespread and grows in various soil conditions, being found on every continent except Antarctica [22], its use in poultry nutrition in Poland and the EU is still not very common. To our knowledge, the effectiveness of DE has not yet been tested as part of a coccidiosis control measure in broilers, with the exception of a study in chickens infected with *E. tenella* where dandelion was part of herbal powder “Shi Ying Zi” consisting of *Cnidium monnieri* (L.) Cuss, *Taraxacum mongolicum* Hand.-Mazz., and sodium chloride [58]. The current study validates the effectiveness of DE in mitigating the effects of *Eimeria* infection in birds. It also confirms the safety of using DE as a feed additive in conjunction with ACV. Importantly, in the current experiment, DE showed no direct oocyst-damaging coccidiocidal effect as indicated in previous studies for oregano, garlic [59], or artemisia, tea tree, thyme, and clove EOs [60], thereby allowing for the continued circulation of vaccine oocysts. The results of this study could support poultry production with reduced or no use of coccidiostats in favor of phytonutrients and/or immunoprophylaxis. However, future studies in large-scale farming conditions are needed to corroborate our results. Also, further in-depth research on the effects of individual selected active substances contained in dandelion would be advisable to determine their exact mode of action in terms of ACV and/or *Eimeria* challenge.

## 5. Conclusions

As revealed in our study, ACV effectively mitigated the negative impacts of homologous infection and improved the final BWG in chickens. DE on its own had a positive effect on the growth performance of birds infected with *Eimeria* in the finisher phase. It reduced PI oocyst shedding, improved intestinal microarchitecture, and positively affected the activity of intestinal microbiota stimulating the cecal production of butyric acid. Furthermore, DE also enhanced the effectiveness of ACV by improving both the humoral immune response and the integrity of the intestinal barrier, as reflected in elevated plasma IgM levels and jejunal TJs proteins profile (ZO-1 and claudin-3), respectively.

## Figures and Tables

**Table 1:** Composition and content of nutrients in basic feed mixtures.

Ingredient [g/kg]	Starter, 1–10 d of Age	Grower 1, 11–19 d of Age	Grower 2, 20–35 d of Age	Finisher, 36–42 d of Age
Wheat	100	100	100	100
Maize	384	404.7	413.9	436.9
Rye	100	100	100	100
Soybean meal	330	300	280	250
Fish meal, 65% CP	20	20	20	20
Rapeseed oil	30	41	52	60
Limestone	12	12	12	12
Sodium chloride	3	3	3	3
Monocalcium phosphate	13	12	11	11
DL-Methionine	2.5	2.3	2.4	2
L-Lysine hydrochloride	1	1.5	1.7	1.6
L-Treonine	0.5	0.5	1	0.5
Vitamin–mineral premix *	4	3	3	3
Analyzed chemical composition (g/kg):				
Dry matter	903	899	899	905
Crude protein	221	215	198	187
Crude fat	51.1	57.8	72.3	78.6
Crude ash	55.3	55.2	51.1	48.2
Crude fiber	29.6	28.3	2.22	2.18
Calcium	9.73	9.70	9.50	7.97
Phosphorus	6.83	6.71	6.41	5.98
Aspartic acid	20.93	19.98	19.94	17.5
Threonine	8.05	7.7	7.91	6.84
Serine	10.13	9.66	10.07	8.64
Glutamic acid	38.28	35.93	35.86	31.57
Proline	12.16	11.7	11.16	10.38
Glycine	9.23	9.2	9	8.12
Alanine	9.68	9.65	10.03	8.8
Valine	9.92	9.47	9.38	8.23
Isoleucine	8.66	8.23	8.18	7.2
Leucine	16.19	15.82	16.47	14.02
Tyrosine	7.27	7.25	7.54	6.11
Phenylalanine	11.42	10.73	10.59	9.32
Histidine	5.25	4.78	4.9	4.36
Lysine	13.24	12.06	11.99	10.53
Arginine	17.7	16.02	15.12	12.28
Cysteine	3.23	2.92	3.03	2.65
Methionine	4.97	4.87	5.04	4.06
Tryptophan	3.08	2.68	2.9	2.29

* Each kilogram of the vitamin-mineral premix contained the following: vitamin A—2,000,000 IU; vitamin D3—500,000 IU; vitamin E—7000 IU; vitamin K3—600 mg; vitamin B1—400 mg; vitamin B2—1400 mg; vitamin B6—1000 mg; vitamin B12—8 mg; Ca-pantothenate—2000 mg; niacin—8000 mg; folic acid—200 mg; biotin—16 mg; choline chloride—29,480 mg; manganese—16,000 mg; zinc—12,000 mg; iron—12,000 mg; copper—3000 mg; iodine—400 mg; selenium—50 mg.

**Table 2:** Effects of experimental factors on the growth performance.

		1–10 d (Starter)			11–20 d (Grower 1)			21–35 d (Grower 2)			36–42 (Finisher)			1–42 d		
Factors		BWG	FCR	FI	BWG	FCR	FI	BWG	FCR	FI	BWG	FCR	FI	BWG	FCR	FI
ACV	DE	[g]	[g/g]	[g]	[g]	[g/g]	[g]	[g]	[g/g]	[g]	[g]	[g/g]	[g]	[g]	[g/g]	[g]
−		305	1.28	389	706	1.45 ^b^	1024 ^b^	1464 ^b^	1.73	2531	623	2.28	1409 ^b^	3096 ^b^	1.74	5379 ^b^
+		307	1.26	386	694	1.56 ^a^	1080 ^a^	1532 ^a^	1.72	2635	660	2.32	1519 ^a^	3193 ^a^	1.76	5626 ^a^
	−	313 ^a^	1.27	395 ^a^	692	1.51	1045	1490	1.73	2580	612 ^b^	2.37	1446	3107	1.76	5468
	+	299 ^b^	1.27	380 ^b^	707	1.50	1059	1506	1.72	2586	670 ^a^	2.22	1483	3182	1.74	5537
−	−	311	1.28	399	692	1.48	1025	1445	1.76	2539	602	2.31	1379	3051	1.75	5348
	+	298	1.27	379	719	1.42	1023	1482	1.70	2524	643	2.25	1439	3142	1.72	5411
+	−	314	1.25	391	693	1.54	1065	1535	1.71	2621	622	2.44	1512	3163	1.77	5589
	+	300	1.27	380	696	1.58	1096	1529	1.73	2649	697	2.19	1526	3222	1.76	5664
SEM		2.07	0.029	2.63	4.08	0.019	11.5	15.4	0.019	35.8	14.6	0.040	20.2	23.9	0.013	53.5
*p*-value																
Effects	ACV	0.536	0.164	0.405	0.120	0.003	0.012	0.027	0.821	0.178	0.178	0.608	0.004	0.036	0.362	0.022
	DE	0.000	0.668	0.001	0.051	0.770	0.484	0.582	0.717	0.928	0.041	0.057	0.274	0.092	0.452	0.209
Interaction	ACV × DE	0.912	0.213	0.267	0.112	0.129	0.408	0.456	0.327	0.774	0.520	0.215	0.496	0.705	0.671	0.888

^a, b^—In each column, mean values not sharing a common superscripted letter are significantly different (*p* ≤ 0.05); ACV—anticoccidial vaccine; DE—dandelion extract; BWG—body weight gain; FI—feed intake; FCR—feed conversion ratio; SEM—standard errors mean.

**Table 3:** *Eimeria* oocyst counts per gram of feces [log10 (OPG + 1)] collected post infection (PI) with *Eimeria* spp. and intestinal lesion scoring at 5 d PI.

Factors		OPG	OPG	OPG	OPG	LS	LS
ACV	DE	5 d PI	6 d PI	7 d PI	15 d PI	Duodenum	Jejunum/Ileum
−		4.41 ^a^	3.74 ^a^	3.52 ^a^	2.77	0.76 ^a^	0.79
+		2.62 ^b^	3.21 ^b^	3.17 ^b^	2.54	0.14 ^b^	0.41
	−	3.62	3.57	3.49 ^a^	3.06 ^a^	0.38	0.74
	+	3.41	3.39	3.20 ^b^	2.25 ^b^	0.53	0.46
−	−	4.29 ^a^	3.80	3.66	3.01	0.70	0.93
	+	4.53 ^a^	3.68	3.39	2.54	0.83	0.65
+	−	2.95 ^b^	3.34	3.32	3.11	0.05	0.55
	+	2.29 ^c^	3.09	3.01	1.96	0.23	0.27
SEM		0.222	0.077	0.066	0.161	0.111	0.101
*p*-value							
Effects	ACV	0.000	0.000	0.001	0.392	0.003	0.059
	DE	0.144	0.081	0.003	0.008	0.404	0.155
Interaction	ACV × DE	0.004	0.504	0.865	0.226	0.873	0.982

^a, b, c^—In each column, mean values not sharing a common superscripted letter are significantly different (*p* ≤ 0.05); ACV—anticoccidial vaccine, DE—dandelion extract, and SEM—standard errors mean.

**Table 4:** Effects of experimental factors on the histometric measurements of the duodenum sampled at 5 d post infection with *Eimeria* spp.

Factors		Longitudinal m. Lamina Thickness [μm]	Circular m. Lamina Thickness [μm]	Submucosa Thickness [μm]	Mucosa Thickness [μm]	Villus Height [μm]	Villus Thickness [μm]	Crypt Depth [μm]	Crypt Width [μm]	Villus Height/Crypt Depth Ratio	Mucosal Surface Absorptive Area [μm^2^]
ACV	DE										
−		61.1	183	35.6	1365	1189	128	112	39.4	12.0	22.5
+		64.1	165	25.7	1441	1268	136	106	41.4	12.3	22.5
	−	68.1	178	27.6	1453	1263	138	119	42.9	10.7	21.9
	+	57.0	169	33.7	1353	1193	127	99	37.9	13.6	23.2
−	−	64.3	185	28.0	1447	1241	129	127	42.4	9.8	22.3
	+	57.9	181	43.2	1283	1137	128	97	36.4	14.2	22.7
+	−	71.9	172	27.2	1460	1286	148	111	43.5	11.5	21.4
	+	56.2	157	24.3	1423	1250	125	100	39.4	13.0	23.7
SEM		2.95	10.4	4.94	34.9	31.8	4.02	5.24	1.40	1.07	0.623
*p*-value											
Effects	ACV	0.604	0.423	0.343	0.273	0.234	0.303	0.549	0.455	0.914	0.981
	DE	0.067	0.661	0.554	0.157	0.285	0.140	0.056	0.084	0.199	0.332
Interaction	ACV × DE	0.416	0.794	0.383	0.360	0.597	0.159	0.361	0.721	0.506	0.465

*p* > 0.05; ACV—anticoccidial vaccine, DE—dandelion extract, and SEM—standard errors mean.

**Table 5:** Effects of experimental factors on the histometric measurements of the jejunum sampled at 5 d post infection with *Eimeria* spp.

Factors		Longitudinal m. Lamina Thickness [μm]	Circular m. Lamina Thickness [μm]	Submucosa Thickness [μm]	Mucosa Thickness [μm]	Villus Height [μm]	Villus Thickness [μm]	Crypt Depth [μm]	Crypt Width [μm]	Villus Height/Crypt Depth Ratio	Mucosal Surface Absorptive Area [μm^2^]
ACV	DE										
−		79.4	184	42.0	1273	1115	87.8 ^b^	141 ^a^	38.4	7.96 ^b^	25.5
+		80.7	165	39.1	1249	1109	102.9 ^a^	119 ^b^	35.7	9.36 ^a^	24.6
	−	88.0	186	38.9	1213	1073	87.0 ^b^	128	35.2	8.48	25.9
	+	72.1	163	42.1	1309	1151	103.7 ^a^	132	38.9	8.84	24.3
−	−	85.3	214	42.8	1212	1069	83.2	139	35.6	7.79	26.1
	+	73.4	154	41.1	1334	1161	92.3	143	41.2	8.14	24.9
+	−	90.7	157	35.1	1214	1077	90.8	118	34.8	9.17	25.6
	+	70.7	172	43.0	1285	1141	115.0	121	36.6	9.54	23.7
SEM		4.73	10.0	2.25	26.7	23.8	3.38	4.44	1.24	0.257	0.556
Significance (*p*-value)											
Effects	ACV	0.890	0.305	0.537	0.653	0.903	0.003	0.015	0.276	0.005	0.447
	DE	0.111	0.241	0.507	0.084	0.122	0.001	0.647	0.146	0.413	0.180
Interaction	ACV × DE	0.673	0.059	0.314	0.629	0.781	0.100	0.965	0.440	0.973	0.751

^a, b^—In each column, mean values not sharing a common superscripted letter are significantly different (*p* ≤ 0.05); ACV—anticoccidial vaccine, DE—dandelion extract, and SEM—standard errors mean.

**Table 6:** Effects of experimental factors on the histometric measurements of the ileum sampled at 5 d post infection with *Eimeria* spp.

Factors		Longitudinal m. Lamina Thickness [μm]	Circular m. Lamina Thickness [μm]	Submucosa Thickness [μm]	Mucosa Thickness [μm]	Villus Height [μm]	Villus Thickness [μm]	Crypt Depth [μm]	Crypt Width [μm]	Villus Height/Crypt Depth Ratio	Mucosal Surface Absorptive Area [μm^2^]
ACV	DE										
−		82.9	201	35.1	845	698	96.1	108 ^b^	37.1	6.51 ^a^	15.8
+		82.2	183	36.5	870	699	92.7	135 ^a^	35.9	5.17 ^b^	16.4
	−	90.6 ^a^	203	37.6	875	707	91.7	125	36.4	5.74	16.5
	+	74.5 ^b^	181	34.0	840	690	97.1	119	36.6	5.94	15.7
−	−	90.5	207	36.5	910	747	99.0	114	38.8	6.56	16.2
	+	75.4	195	33.6	780	650	93.2	102	35.3	6.45	15.4
+	−	90.8	200	38.7	840	668	84.4	136	33.9	4.92	16.7
	+	73.7	167	34.4	901	730	101.0	135	37.8	5.42	16.0
SEM		3.45	8.70	2.02	23.1	22.6	3.63	4.17	0.925	0.230	0.482
*p*-value											
Effects	ACV	0.912	0.324	0.733	0.563	0.991	0.641	0.000	0.508	0.002	0.582
	DE	0.022	0.210	0.406	0.425	0.701	0.464	0.293	0.912	0.595	0.463
Interaction	ACV × DE	0.873	0.573	0.870	0.054	0.097	0.140	0.346	0.055	0.411	0.996

^a, b^—In each column, mean values not sharing a common superscripted letter are significantly different (*p* ≤ 0.05); ACV—anticoccidial vaccine, DE—dandelion extract, and SEM—standard errors mean.

**Table 7:** Quantitative analysis of the intensity of zonula occludens-1 and claudin-3 immunoreaction (IR) of the jejunum of broiler chickens at 5 days post infection.

Factors		ZO-1, Villi	Claudin-3, Crypts
ACV	DE	(OD)	(OD)
−		0.254 ^b^	0.238 ^a^
+		0.339 ^a^	0.181 ^b^
	−	0.302	0.216
	+	0.291	0.203
−	−	0.301 ^b^	0.223 ^ab^
	+	0.208 ^c^	0.253 ^a^
+	−	0.304 ^b^	0.210 ^b^
	+	0.373 ^a^	0.153 ^c^
SEM		0.016	0.010
	*p*-value		
Effects	ACV	0.000	0.000
	DE	0.529	0.210
Interaction	ACV × DE	0.000	0.001

^a, b, c^—In each column, mean values not sharing a common superscripted letter are significantly different (*p* ≤ 0.05); ACV—anticoccidial vaccine, DE—dandelion extract, ZO-1—zonula occludens-1, OD—optical density, and SEM—standard errors mean.

**Table 8:** Effects of experimental factors on plasma immunoglobulin, cytokines, and acute phase proteins levels of broiler chickens at 5 d post infection with *Eimeria* spp.

Factors		IgY	IgM	IgA	TNF-α	IL-1β	IL-6	Cp	Fb
		[mg/mL]	[mg/mL]	[µg/mL]	[pg/mL]	[pg/mL]	[pg/mL]	[µg/mL]	[mg/mL]
ACV	DE								
−		10.1	6.56 ^b^	465	81.3	60.5	114.9 ^a^	77.9	5.28
+		11.1	9.37 ^a^	430	68.2	44.3	92.6 ^b^	67.5	3.86
	−	10.7	6.59 ^a^	419	80.1	64.1	106.6	72.2	4.86
	+	10.5	9.34 ^b^	476	69.3	40.7	101.0	73.2	4.27
−	−	12.2 ^ab^	6.39 ^b^	411	86.1	79.5	115.9	80.8	5.63
	+	8.12 ^b^	6.72 ^b^	518	76.5	41.4	114.0	75.0	4.92
+	−	9.31 ^ab^	6.79 ^b^	426	74.1	48.6	97.2	63.6	4.09
	+	12.9 ^a^	11.96 ^a^	433	62.2	40.0	88.0	71.4	3.62
SEM		0.803	0.707	30.2	4.78	6.74	4.61	3.39	0.398
		*p*-value							
Effects	ACV	0.511	0.014	0.580	0.186	0.208	0.015	0.142	0.087
	DE	0.880	0.016	0.376	0.273	0.076	0.505	0.881	0.459
Interaction	ACV × DE	0.018	0.031	0.432	0.901	0.247	0.664	0.327	0.881

^a, b^—In each column, mean values not sharing a common superscripted letter are significantly different (*p* ≤ 0.05); ACV—anticoccidial vaccine, DE—dandelion extract, IgY—immunoglobulin Y, IgM—immunoglobulin M, IgA—immunoglobulin A, TNF-α—tumor necrosis factor-α, IL-1β—interleukin-1β IL-6—interleukin-6, Cp—ceruloplasmin, Fb—fibrinogen, and SEM—standard errors mean.

**Table 9:** Biochemical parameters of chicken blood sampled at 5 d PI.

Factors		GLU	TG	TC	TP	ALT	AST	ALP	LDH
ACV	DE	[mg/dL]	[mg/dL]	[mg/dL]	[g/dL]	[U/L]	[U/L]	[U/L]	[U/L]
−		228	38.8	135 ^b^	2.58 ^b^	10.61 ^a^	241	3748	3041
+		227	43.2	150.9 ^a^	2.85 ^a^	6.68 ^b^	251	4300	3008
	−	225	40.1	140.5	2.64	9.45	240	4132	2875
	+	230	41.9	145.4	2.79	7.83	252	3917	3174
−	−	224	40.6	139.4 ^b^	2.66 ^b^	12.54 ^a^	239	3757	2789
	+	231	37	130.6 ^b^	2.50 ^b^	8.67 ^b^	243	3739	3294
+	−	225	39.6	141.6 ^ab^	2.62 ^b^	6.36 ^b^	242	4507	2962
	+	229	46.8	160.2 ^a^	3.07 ^a^	7.00 ^b^	261	4094	3053
SEM		3.30	1.91	3.79	2.58	0.728	7.12	212	172
		*p*-value							
Effects	ACV	0.944	0.257	0.022	0.003	0.002	0.501	0.223	0.927
	DE	0.447	0.637	0.445	0.069	0.139	0.443	0.628	0.424
Interaction	ACV × DE	0.834	0.168	0.044	0.001	0.045	0.633	0.657	0.577

^a, b^—In each column, mean values not sharing a common superscripted letter are significantly different (*p* ≤ 0.05); ACV—anticoccidial vaccine, DE—dandelion extract, GLU—glucose, TG—triacylglycerol, TC—total cholesterol, TP—total protein, ALT—alanine aminotransferase, AST—aspartate aminotransferase, ALP—alkaline phosphatase, LDH—lactate dehydrogenase, and SEM—standard errors mean.

**Table 10:** Effects of experimental factors on the volatile fatty acid concentrations in the cecal digesta of broiler chickens at 42 d of age.

Factors		Acetic Acid	Propionic Acid	Isobutyric Acid	Butyric Acid	Isovaleric Acid	Valeric Acid	BCFAs	Total VFA
		[µmol/g]	[µmol/g]	[µmol/g]	[µmol/g]	[µmol/g]	[µmol/g]	[µmol/g]	[µmol/g]
ACV	DE								
−		83.3	7.02	1.21	13.2	1.47	1.45	2.69	107.6
+		77.3	7.27	1.07	11.8	1.27	1.27	2.33	99.9
	−	78.5	7.46	1.10	11.0 ^b^	1.30	1.30	2.40	100.6
	+	82.1	6.83	1.18	14.0 ^a^	1.44	1.42	2.62	107.0
−	−	80.5	6.86	1.14	11.8	1.39	1.40	2.53	103.1
	+	86.2	7.18	1.29	14.5	1.56	1.50	2.84	112.2
+	−	76.4	8.06	1.05	10.1	1.22	1.20	2.27	98.1
	+	78.1	6.48	1.08	13.5	1.32	1.34	2.40	101.8
SEM		3.42	0.431	0.053	0.748	0.070	0.055	0.120	4.34
		*p*-value							
Effects	ACV	0.393	0.773	0.166	0.354	0.153	0.109	0.150	0.391
	DE	0.605	0.477	0.424	0.048	0.330	0.292	0.359	0.478
Interaction	ACV × DE	0.774	0.284	0.592	0.810	0.811	0.839	0.708	0.763

^a, b^—In each column, mean values not sharing a common superscripted letter are significantly different (*p* ≤ 0.05); ACV—anticoccidial vaccine, DE—dandelion extract, BCFAs—branched-chain fatty acids, VFA—volatile fatty acids, and SEM—standard errors mean.

## Data Availability

The data presented in this study are available within the article.

## Supplementary Materials

The following supporting information can be downloaded at: https://www.mdpi.com/article/10.3390/life13091927/s1, Figure S1: Representative images of the immunohistochemical reactions of zonula occludens-1 and claudin-3 carried out on formaldehyde-fixed sections from the jejunum of broiler chickens sampled at 5 d post challenging with *Eimeria* spp.; ACV vaccination at 1 d of age with anticoccidial vaccine and DE dietary supplementation with *Taraxacum officinale* (dandelion) extract. Examples of the reaction are marked with white arrows. All the scale bars represent 100 μm.
