# Supplementary material for: Effects of Anticoccidial Vaccination and Taraxacum officinale Extract on the Growth Performance, Biochemical Parameters, Immunity, and Intestinal Morphology of Eimeria-Challenged Chickens
Source: Life (Basel). 2023 Sep 17;13(9):1927. doi: 10.3390/life13091927 (PMC10532845; doi:10.3390/life13091927)
Supplement: Supplementary file 1 [file life-13-01927-s001.zip › life-2574088-supplementary.pdf]

Supplementary Material

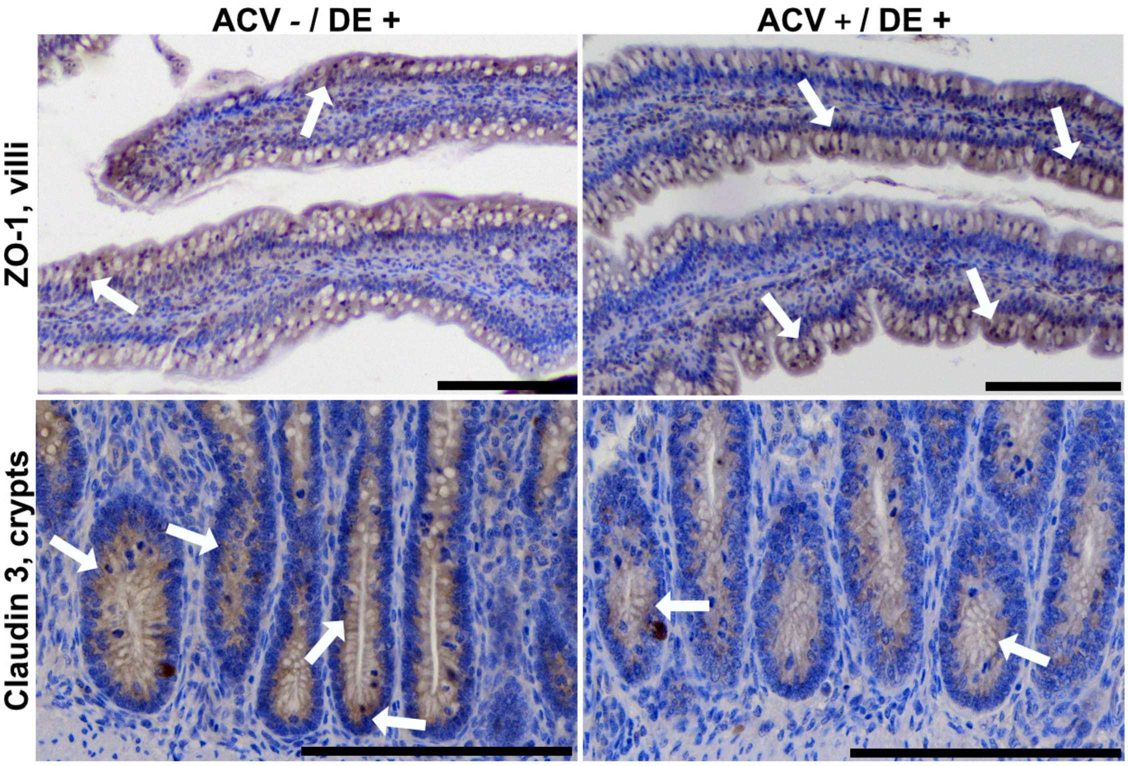

Figure S1. Representative images of the immunohistochemical reactions of zonula occludens-1 and claudin -3 carried out on formaldehyde-fixed sections from the jejunum of broiler chickens sampled at 5 d post challenging with *Eimeria* spp.; ACV—vaccination at 1 d of age with anticoccidial vaccine and DE—dietary supplementation with *Taraxacum officinale* (dandelion) extract. Examples of the reaction are marked with white arrows. All the scale bars represent 100 μm.
